# FHBMarkerDb: unifying genomic markers and functional annotations for durable FHB resistance in major cereals

**DOI:** 10.3389/fpls.2026.1846092

**Published:** 2026-07-16

**Authors:** Ankita Mohapatra, Yuvraj Singh, Divya Sharma, Dwijesh Chandra Mishra, Anjan Kumar Pradhan, Girish Kumar Jha, Amit Kumar Singh, Rakesh Singh, G P Singh, Neeraj Budhlakoti, Sundeep Kumar

**Affiliations:** 1 Division of Genomic Resources, Indian Council of Agricultural Research (ICAR) - National Bureau of Plant Genetic Resources, New Delhi, India; 2 Division of Agricultural Bioinformatics, Indian Council of Agricultural Research (ICAR) - Indian Agricultural Statistics Research Institute, New Delhi, India

**Keywords:** database, FHB, FHBDb, FHBMarkerDb, MTAS

## Abstract

Fusarium Head Blight (FHB), caused by multiple *Fusarium* species, is a major disease of cereal crops worldwide, resulting in significant yield losses and grain contamination with mycotoxins. Although many genetic and genomic studies have identified markers, quantitative trait loci (QTL), and candidate genes for FHB resistance, this information remains dispersed across multiple sources, limiting its use in breeding and research. To overcome this, we developed the Fusarium Head Blight Marker Database (FHBMarkerDb), a centralized, curated database that compiles comprehensive genomic resources on FHB resistance available in the public domain. FHBMarkerDb integrates publicly available marker trait associations (MTAs), candidate genes, and functional annotations for four major cereal crops- wheat, barley, maize, and oats within a single platform, enabling cross-species comparison and comparative genomics analyses. The database covers key FHB-related traits, including disease incidence, disease severity, disease spread, and mycotoxin accumulation, providing a holistic view of resistance mechanisms. Further, the user-friendly interface provides flexibility of efficient searching, browsing, and retrieval of markers, chromosomes, traits, genes and their functions. We believe the developed resource will empower researchers, breeders, and students to identify promising resistance loci and further accelerate genomics-assisted breeding. The database is freely accessible at https://nbpgr.org.in/FHBDb/index.php.

## 1 Introduction

FHB is a globally devastating disease primarily affecting small grain cereals such as wheat, barley, maize, and oats, caused mainly by *Fusarium graminearum* and related species. The disease causes substantial yield losses, degrades grain quality, and contaminates crops with harmful mycotoxins like deoxynivalenol (DON), which poses serious health risks to humans and livestock and results in strict regulatory limits on grain use (Islam, 2022; Alisaac and Mahlein, 2023; Mir et al., 2023). FHB causes substantial yield losses worldwide, exceeding a billion dollars annually due to yield reduction, steep price discounts and increased production costs due to integrated management practices including resistant cultivars, fungicides, and cultural controls (Dahl and Wilson, 2018; Wilson et al., 2018). Major epidemics, such as those in North America during the1990s, have highlighted the severe impact of FHB on wheat and barley production, prompting coordinated research efforts to develop improved management strategies (McMullen et al., 2012; Powell and Vujanovic, 2021). The disease’s economic burden extends beyond yield loss to include market restrictions and risk premiums for producers, emphasizing the importance of continued breeding and integrated pest management (Dahl and Wilson, 2018; Wilson et al., 2018). Understanding the epidemiology, host range, and economic impact of FHB is critical for safeguarding cereal crop production and food safety globally.

Despite advances in chemical fungicides and breeding technologies that have produced moderately resistant cultivars, FHB remains a persistent challenge for cereal production and productivity worldwide. This difficulty arises due to disease causation by a complex of Fusarium species, which hinders the development of effective control measures against multiple pathogenic strains simultaneously. Further, diversity within the Fusarium species complex complicates resistance breeding and management, as they may vary in geographic adaptation and mycotoxin profiles, which requires integrated approaches for effective control (Dweba et al., 2017; Islam, 2022; Alisaac and Mahlein, 2023). Additionally, limited understanding of the pathogen’s biology and quantitative nature of FHB resistance further limits the rapid genetic improvement of resistant cultivars (Dweba et al., 2017). Environmental factors such as warm and wet conditions during flowering time, further intensify disease outbreaks, reduce the efficacy of current control measures. Therefore, overcoming these constraints requires interdisciplinary strategies combining genetic, chemical, cultural, and biological controls tailored to the complex Fusarium pathosystem (Legrand et al., 2019).

Over time, molecular markers, QTL mapping and genomics approaches have been extensively used to improve FHB resistance in wheat, barley, oats, and maize. In wheat, numerous QTLs have been identified on chromosomes such as 1B, 3B (notably Fhb1), 5B, 6A, and 7A, explaining significant phenotypic variation to FHB resistance. Further, marker-assisted selection (MAS) using Kompetitive Allele-Specific PCR (KASP) markers developed from these QTLs accelerates breeding of resistant cultivars (Su et al., 2018; Ren et al., 2025; Song et al., 2025; Neupane et al., 2025; Zhu et al., 2025). Meta-QTL analyses integrating multiple studies have refined these loci and identified candidate genes involved in resistance mechanisms, supporting molecular breeding platforms (Zheng et al., 2021). Although barley, oats, and maize, are less extensively studied than wheat, similar genomic approaches have identified resistance loci and markers in relation to FHB. However, due to the complexity of Fusarium species and host-pathogen interactions, further research efforts are required to develop robust markers and QTLs for underlying crops (Asiedu and Miedaner, 2025; Sallam et al., 2024; Akohoue and Miedaner, 2022; Cao et al., 2022; Santiago et al., 2020; Lanubile et al., 2017). For example, barley shares resistance mechanisms with wheat, with breeding efforts targeting stable QTLs and validated markers for MAS, while maize research priorities detoxification pathways and resistance to mycotoxin accumulation. Overall, integrating genomics, markers, and QTL information across these cereals is essential for the development of durable FHB resistance, though wheat leads as most advanced model, featuring well-characterized QTLs and markers ready for breeding programs (Su et al., 2018; Ren et al., 2025; Song et al., 2025; Neupane et al., 2025; Zheng et al., 2021; Zhang et al., 2024).

Over time, QTLs and marker information related to FHB have rapidly accumulated, but remains dispersed across individual studies, crop-specific databases, and supplemental resources. This further hinders their effective use in comparative genomics and breeding programme (Kazan and Gardiner, 2018; Ma et al., 2020; Buerstmayr et al., 2020; Nannuru et al., 2022; Viviani et al., 2025). Even in wheat, comprehensive efforts required “assembling the QTLome” through exhaustive literature curation, as data spanned over 70 publications and heterogeneous marker systems (Venske et al., 2019). Durum wheat and international wheat improvement programs face similar needs for systematic aggregation and uniform presentation of FHB loci and markers (Ma et al., 2020; Buerstmayr et al., 2020; Haile et al., 2023; Viviani et al., 2025). This fragmentation presents a major challenge for the researchers and breeders seeking to identify conserved resistance loci, understand shared biological pathways, or deploy validated markers in breeding programs. Although genomic repositories exist, none offer a comprehensive, trait-focused, cross-species platform that integrates genetic loci with gene function, ontology, and pathway information.

To address this gap, we developed FHB marker database (FHBMarkerDb), a comprehensive and well curated database comprising gene ontology annotations, candidate genes, chromosomal locations, FHB-associated SNPs, QTLs, and pathway correlations from cereal including wheat, barley, maize, and oats. FHBMarkerDb accelerates the identification and deployment of resistance loci for breeding FHB-resistant varieties, supports candidate gene prioritization, and enables cross-species comparisons, all through a single, intuitive platforms that unifies dispersed genomic and trait data.

## 2 Materials and methods

### 2.1 Data collection and curation

The core data for FHBMarkerDb was manually curated from the peer-reviewed scientific literature available on the web. The curation process focused on identifying MTAs and QTL reported for FHB resistance and related traits such as yield under disease pressure, mycotoxin accumulation, and heading date across major cereal crops, including wheat, barley, maize, and oats. This exhaustive effort involved systematically reviewing hundreds of studies to extract standardized metadata on genetic markers (e.g., SNPs, SSR), effect sizes, chromosomal positions, underlying candidate genes, etc., ensuring high-quality, reliable inputs for downstream breeding applications.

#### 2.1.1 MTA/QTL extraction from published literature

We systematically surveyed published research articles, QTL mapping studies, genome-wide association studies (GWAS), and meta-analyses-based studies. Literature was searched using databases such as PubMed, Google Scholar, and Web of Science with keywords including “Fusarium Head Blight,” “FHB resistance,” “QTL,” “GWAS,” “marker-trait association,” “mycotoxin (DON) resistance,” along with crop names (wheat, maize, oats, barley). Studies with statistically significant MTAs/QTLs (−log_10_(p) ≥ 3) were included, while studies with insufficient data were excluded. To ensure data quality, all selected studies were carefully reviewed and the extracted information was cross-checked for accuracy. For each reported significant MTAs/QTL (based on study-specific statistical thresholds), the following key information was extracted:

- Crop species: Primary host crop (e.g., Triticum aestivum (bread wheat), Triticum durum (durum wheat), Zea mays (maize), Hordeum vulgare (barley), Avena sativa (oats)) (**Table 1**).
- FHB-related trait: The specific phenotypic trait measured [e.g., FHB severity, Fusarium Damaged Kernels (FDK%), deoxynivalenol (DON) accumulation, resistance type (Type I, II, etc.)] (**Table 2**).
- Genetic Marker: The name and type of the associated marker (e.g., SSR, QTL, SNP).
- Genomic Position: Included the chromosome and physical/genetic map coordinates, wherever available.

**Table 1:** Details of curated data available in FHBMarkerDb.

Crop	Scientific name	FHB-associated traits
Bread Wheat	*Triticum aestivum*	FHB Severity(SEV/FHB), Deoxynivalenol (DON) content, FHB incidence (INC), Proline(PRO) content, Index of Severity and Deoxynivalenol(ISD), Plant height (PH/HT/PHRating/PHT/PLHT), Days to Maturity (MAT),Heading Date(HD), Yellow Pigment(YP), FHB Index(IND), Days to Anthesis(DTA), Fusarium Damaged Kernels (FDK), Fourier Transform Method(FTM), Not Otherwise Specified (NOS), Percentage of Symptomatic Spikelets(PSS), Thousand Grain Weight (TGW), Incidence, Severity, and Kernel Damage Index (ISK)
Durum Wheat	*Triticum durum*	FHB Severity, Deoxynivalenol (DON) content, FHB incidence (INC), Proline (PRO) content, Index of Severity and Deoxynivalenol (ISD), FHB Visual Rating Index (VRI), Plant height (PH/HT), Days to Maturity (MAT), Heading Date (HD), Yellow Pigment (YP), FHB Index (IND), Days to Anthesis (DTA), Fusarium Damaged Kernels (FDK)
Maize	*Zea mays*	Gibberella Ear Rot Severity (SEV), DON contamination, Fusarium Head Blight Spread Resistance (FSR), Gibberella Ear Rot (GER), Fusarium Ear Rot (FER)
Barley	*Hordeum vulgare*	FHB Severity(FHB/SEV), Deoxynivalenol (DON) content, Kernel Infection, Row Type (RT), Kernel Discoloration (KD), Days To Heading (DTH), Plant height (PH), Number of Scabbed Spikelets per spike (NSS), Spike Length (SL), Grain Yield(GY), length of the main stem (Lst), Hectoliter Kernel Number (HLKn), Grain Weight per Spike (GWS), Heading Date (HD), weight of healthy kernels(HLKw), Fusarium Head Blight Index (FHBi), Thousand Grain Weight (TGW), Fusarium-damaged kernels number (FDKn), Fusarium-damaged kernels weight (FDKw)
Oats	*Avena sativa*	FHB Severity (FHB/SEV), Deoxynivalenol (DON) content

**Table 2:** Crop-wise summary statistics of FHBMarkerDb for different parameter (i.e. no. of studies, MTAs, candidate genes, GO terms, pathways).

Crop	Number of curated studies	Number of MTAs	Number of annotated candidate genes	Number of unique GO terms	Number of pathway involved
*Triticum aestivum* (Bread Wheat)	16	1904	853	543	119
*Triticum durum* (Durum Wheat)	3	1839	1803	41	18
*Zea mays* (Maize)	7	625	584	0	0
*Hordeum vulgare* (Barley)	3	184	18	10	0
*Avena sativa* (Oats)	2	26	0	0	0

This structured curation ensures comprehensive, standardized data for robust downstream analyses.

#### 2.1.2 Candidate gene identification and functional annotation

For MTAs anchored to reference genomes, we identified putative candidate genes within or proximal to the associated genomic intervals. The functional annotation for these genes were enriched through a multi-source bioinformatics pipeline:

- Gene Ontology (GO): Standardized GO terms (Biological Process, Molecular Function, Cellular Component) retrieved using platforms like DAVID, ShinyGO, and Ensembl Plants crop portals.
- Pathway Analysis: Biochemical and signaling pathways involvement assessed through Kyoto Encyclopedia of Genes and Genomes (KEGG) database and ShinyGO modules, highlighting genes in defense-related processes (e.g., phenylpropanoid biosynthesis, hormone signaling).

This approach provides a deeper biological context for prioritizing resistance loci in breeding programs.

#### 2.1.3 Data integration and standardization

All curated data were integrated into a structured relational schema, with crop and trait names standardized using controlled vocabularies (e.g., ontology terms from Gramene and Ensembl Plants). Genomic coordinates were harmonized to latest reference genome assemblies wherever possible, enabling seamless cross-study and cross-species comparisons. This rigorous process resulted in a comprehensive resource that links genetic markers, genomic regions, candidate genes, and functional annotations, empowering FHB research and breeding across multiple cereal crops.

### 2.2 Database development: design & implementation

#### 2.2.1 Design philosophy & architecture

FHBMarkerDb is a centralized, user-friendly database that consolidates and organizes genomic information related to FHB resistance from diverse crop studies, enabling researchers, breeders, and students to easily access, compare, and use this information. The database is built using a standard XAMPP server, integrating MySQL for data storage, PHP for dynamic scripting, and Apache as the web server. It employs a three-tier relational database structure, ensuring straightforward maintenance and efficient handling of large datasets. A standout capability of FHBMarkerDb is the integration of genomic data from four major cereal- wheat, barley, maize, and oat within a single platform. This facilitates cross-species comparisons of genes and markers, accelerating comparative genomics and the identification of conserved resistance mechanisms.

#### 2.2.2 Core technical implementation

Technology Stack: FHBMarkerDb is built on the XAMPP stack (Apache, MySQL, and PHP), paired with a front-end of HTML5, CSS3, and JavaScript. This open-source foundation ensures cost-effective deployment and strong community support.

MySQL efficiently stores vast biological datasets in a normalized relational structure, with PHP handling user requests to query and render data dynamically and displaying on the screen, while Apache delivers content to users’ browsers.

Database Schema: Implements a normalized relational structure in MySQL with following key components:

1. Primary Entities: Hierarchical structure linking species, chromosomes, and markers.
2. Annotation Layers: Genes, Gene Ontology terms, and biochemical pathways.
3. Association Tables: Marker-trait relationships, gene-function links, and pathways connections.

This design enables efficient data retrieval, cross-referencing, and expansion for ongoing and future FHB research and development.

#### 2.2.3 Performance & security design

FHBMarkerDb optimizes performance through composite indexing on frequently queried columns such as species, chromosome, and genomic position. Application-level caching handles reference data efficiently, while optimized JOIN operations support complex biological queries without compromising speed.

Security features include parameterized queries to block SQL injection, role-based access control for different user types, enforce HTTPS for data transmission, and regular security audits of the XAMPP components. This layered approach ensures robust protection for sensitive genomic data.

## 3 Results and discussion

### 3.1 Database content and data types

**Figure 1:**
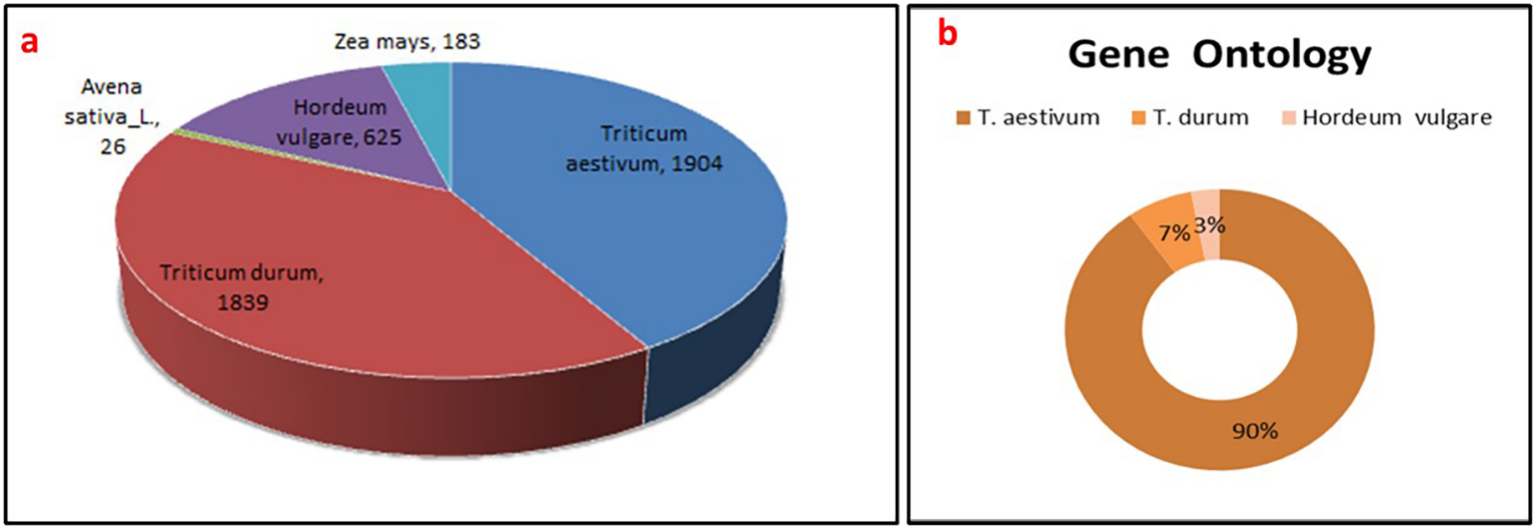
**(A)** Crop-wise distribution of curated information available in FHBMarkerDb across the cereal crops **(A)** MTAs. **(B)** GO annotations.

FHBMarkerDb is a manually curated, multi-crop genomic resource that integrates MTAs, candidate genes, GO annotations, and pathway information in relation to FHB resistance (**Table 1**). The current version of the database contains 4,578 MTAs derived from 31 peer-reviewed studies, covering five cereal species: bread wheat, durum wheat, maize, barley, and oats. Coming to the individual records, Bread wheat contributed the highest number of MTAs (1,904), followed by durum wheat (1,839), maize (625), barley (184), and oats (26) (**Table 2**; **Figures 1a, b**). Candidate gene mining within genome-anchored loci identified 3,258 annotated genes, with the majority identified in wheat. Functional enrichment analysis revealed 543 GO terms and 119 pathways for bread wheat, and 41 GO terms and 18 pathways for durum wheat. In contrast, limited functional annotations in maize, barley, and oats reflect fewer genome-resolved loci and functional studies in these crops. Notably, the enrichment analysis results have been further discussed to highlight key biological processes and signaling pathways potentially regulating FHB resistance, including defense response, hormone-mediated signaling, and stress-related metabolic pathways, thereby providing insights into the molecular regulatory networks underlying resistance mechanisms.

**Figure 2:**
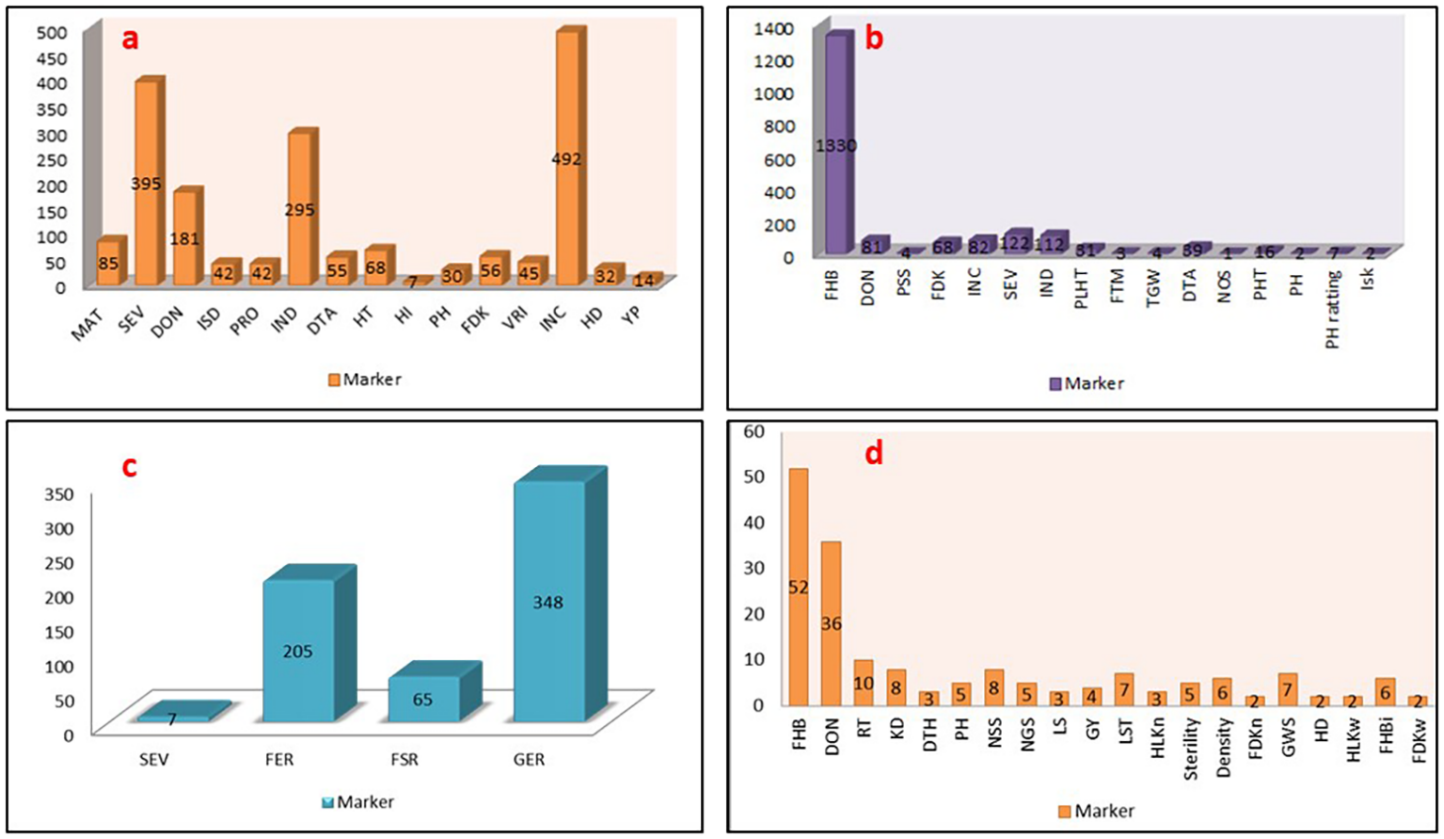
Trait-wise distribution of curated MTAs across cereal crops in FHBMarkerDb. **(A)** *Triticum aestivum* (bread wheat); **(B)** *Triticum durum* (durum wheat); **(C)** *Zea mays* (maize); and **(D)** *Hordeum vulgare* (barley).

The crop-wise trait distribution highlights differences in resistance-associated traits among cereals (**Figures 2A–D**). In addition, agronomic traits such as plant height, heading date, and spike morphology were included due to their known association with disease escape and resistance (Choo et al., 2004; Xiaoyu et al., 2014). This multi-trait and multi-species information framework enables users to explore the complex genetic architecture of FHB resistance and assess pleiotropic or linked effects. Individual database entry provides standardized information on crop species, chromosome position, marker type, associated trait, candidate genes, and functional annotations. By integrating marker–trait–gene relationships, FHBMarkerDb enables users to move from statistical associations to biological interpretation and insights, facilitating candidate gene prioritization and functional validation studies.

### 3.2 Design and implementation of FHBMarkerDb

**Figure 3:**
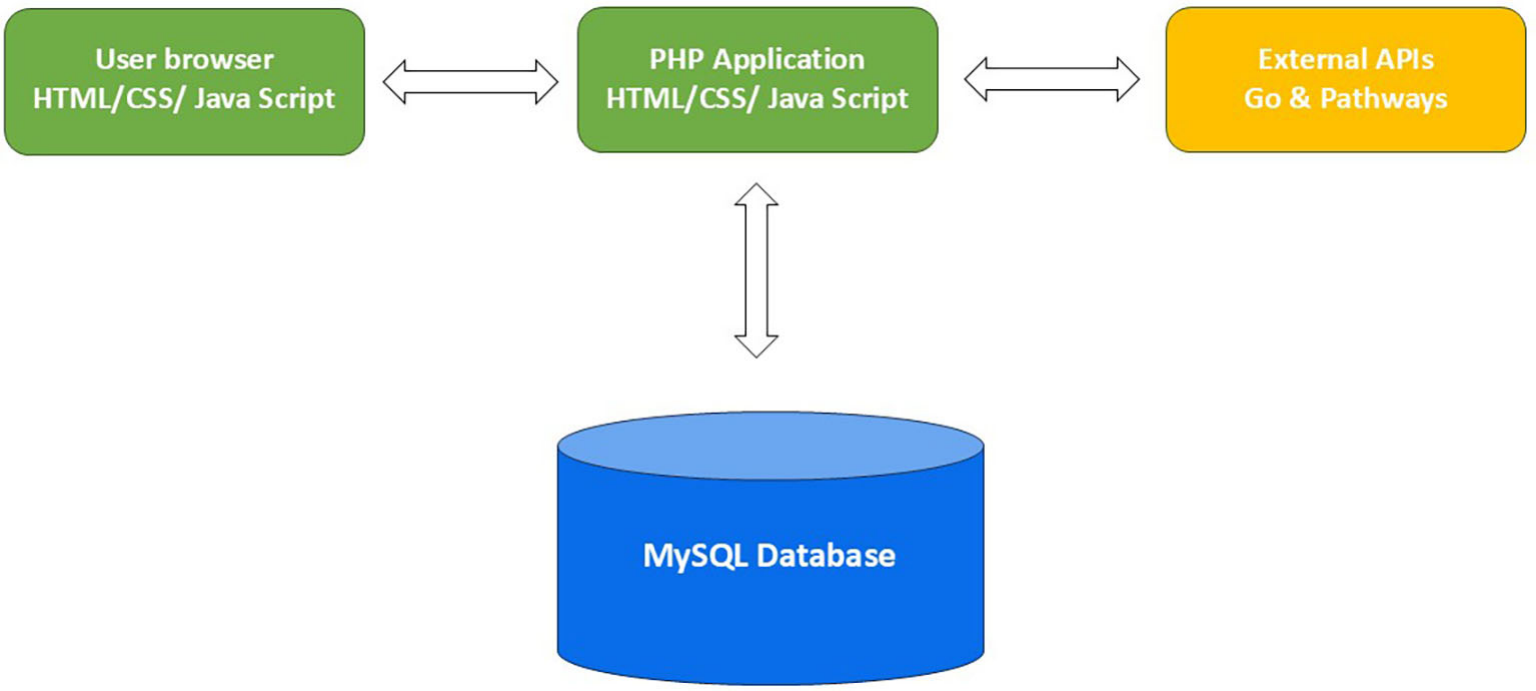
Three-tier system architecture used for the development of FHBMarkerDb.

**Figure 4:**
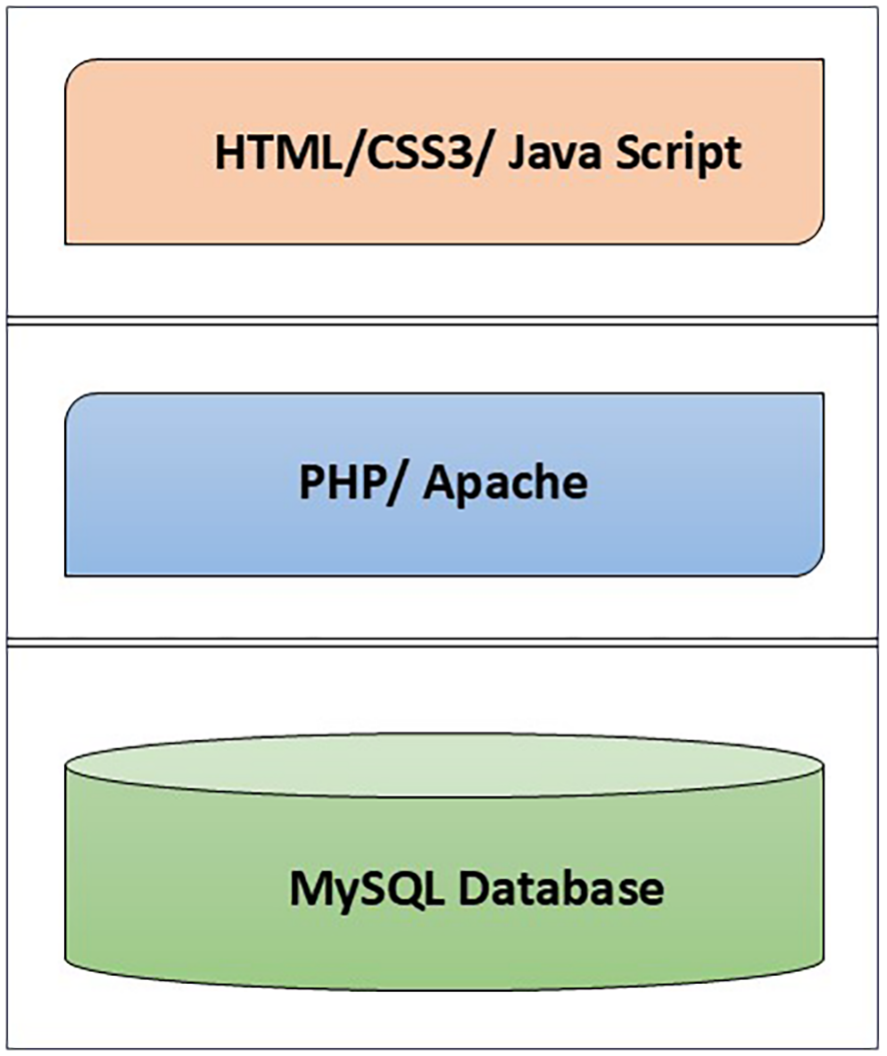
Technology stack of FHBMarkerDb including the web interface, backend, and MySQL database.

FHBMarkerDb was developed using a three-tier architecture comprising the client interface, application layer, and relational database, ensuring modularity and efficient data management (**Figure 3**). The system was implemented on the XAMPP platform, which integrates Apache as the web server, MySQL for structured data storage, and PHP for server-side processing. The front-end interface was built using HTML5, CSS3, JavaScript, and Bootstrap to provide a responsive and user-friendly browsing experience. The overall technology stack of FHBMarkerDb is presented in **Figure 4**.

**Figure 5:**
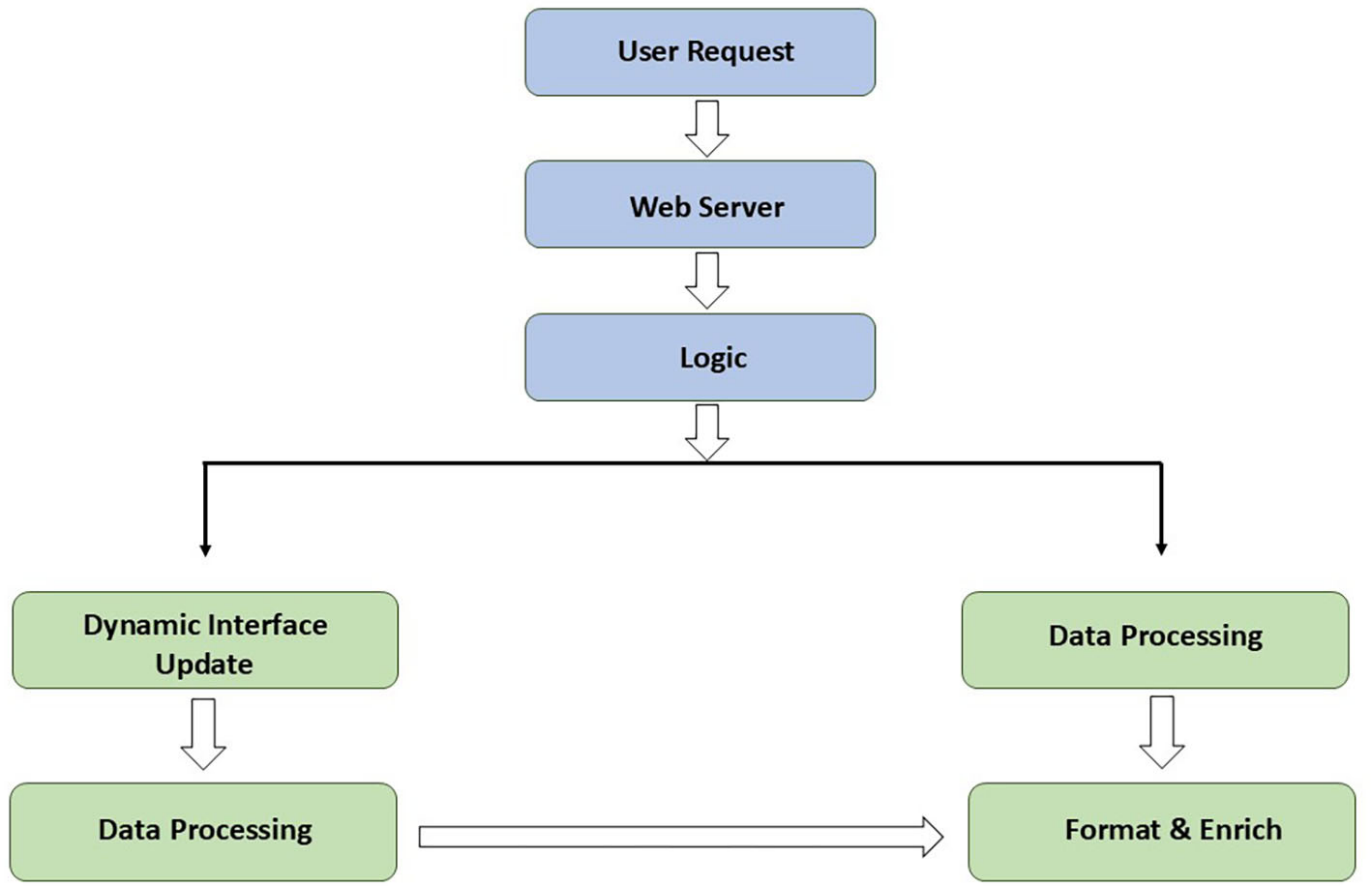
Detailed workflow of FHBMarkerDb illustrating the flow from user request through the web server and application.

The underlying database schema follows a normalized relational structure with a hierarchical organization of species → chromosomes → markers, linked to annotation tables containing genes, GO terms, and pathways. Association tables connect markers with traits and candidate genes, enabling efficient retrieval of biologically meaningful relationships. To improve performance, composite indexing was applied to the frequently queried fields such as species, chromosome, and genomic position. Optimized JOIN operations and caching of reference tables reduced query execution time during testing. The system also incorporates parameterized queries to prevent SQL injection and role-based access control for secure data management. The overall workflow of FHBMarkerDb, including literature curation, data standardization, candidate gene mining, functional annotation, database integration, and web-based data retrieval, is presented in **Figure 5**.

### 3.3 Data querying, interface, and database applications

The FHBMarkerDb web interface provides multiple browsing and search functionalities to facilitate efficient data access. Users can query the database by crop, chromosome, marker name, trait, or candidate gene. The output pages display detailed information, including genomic coordinates, associated traits, linked genes, GO annotations, and pathway involvement.

**Figure 6:**
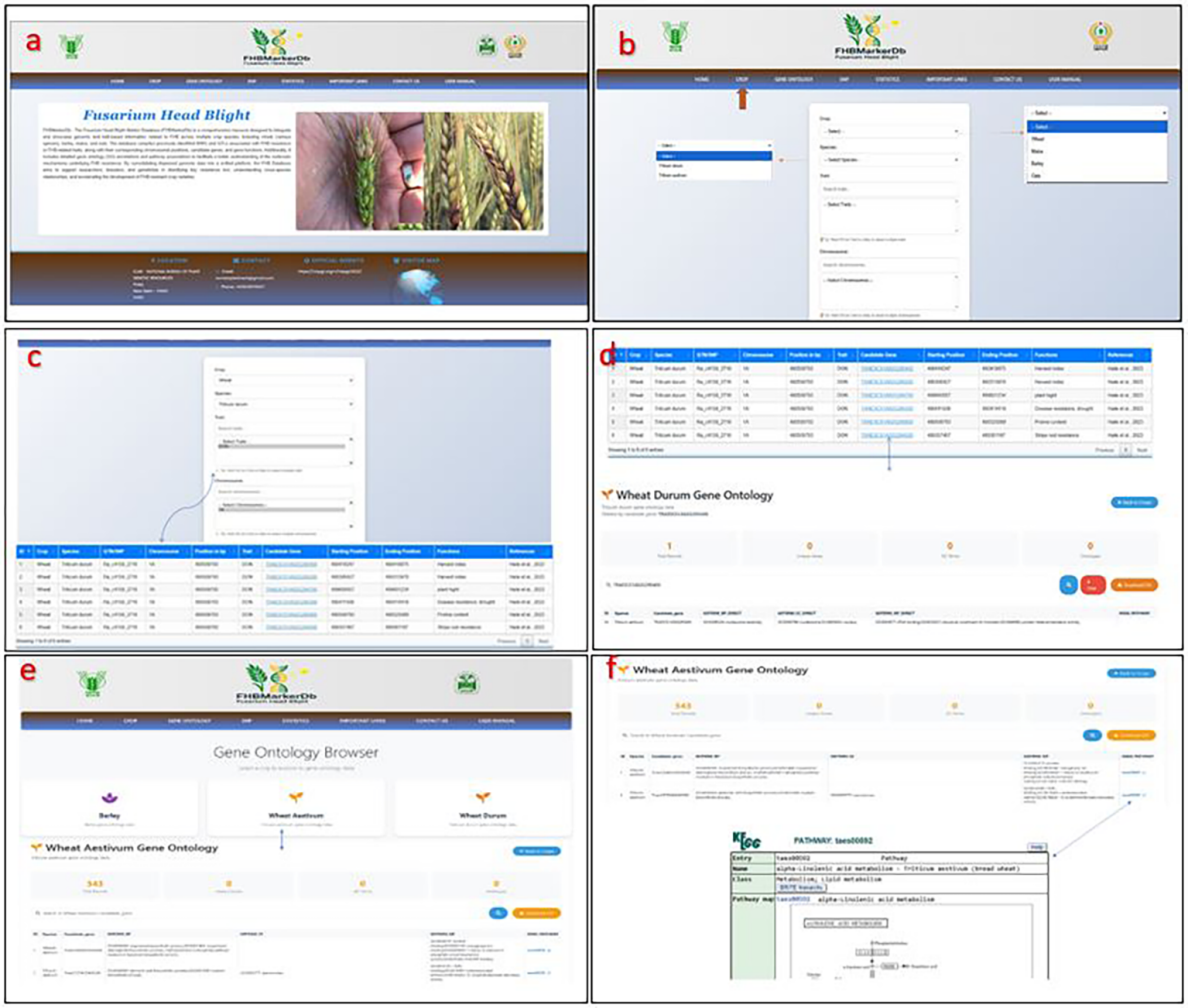
Showcases the diverse features and utilities of FHBMarkerDb through the illustrating screenshots highlighting key interface elements, search functionalities, and data visualization **(A–F)**.

The browse module allows users to explore data in a catalog-based format, while the search module enables customized queries for specific markers or genomic regions. Integrated marker-trait-gene views support rapid identification of resistance loci and functional candidates. The Gene Ontology section provides categorized functional information, and pathway links highlight genes involved in defense-related biological processes. Screenshots of the user interface (**Figures 6a–f**) demonstrate the step-wise selection of parameters, retrieval of marker-associated information, and visualization of functional annotations. These features enable comparative analysis across crops and facilitate the identification of conserved resistance regions. Apart from that a handy user guide is available in the form of “user manual” menu, providing clear guidance to ensure smooth navigation and effective use of FHBMarkerDb’s features.

### 3.4 Application case studies

To demonstrate the practical utility of FHBMarkerDb, representative case studies were performed to illustrate its use in exploring marker trait gene relationships in FHB resistance. In first example, querying a wheat MTA allowed retrieval of linked genomic regions and identification of candidate genes, which showed functional enrichment in defense response and hormone mediated signaling pathways (**Supplementary Figure 1**). In another example, cross-crop comparison of resistance-associated loci enabled identification of conserved candidate genes across cereals, with pathway information indicating involvement in key regulatory processes such as stress response and plant–pathogen interaction networks. These examples highlight how FHBMarkerDb facilitates the transition from marker level associations to biological interpretation and candidate gene prioritization.

### 3.5 Comparison with existing databases

WheatQTLdb is a broad repository covering QTLs across multiple traits in wheat, including morphological, physiological, and stress-related traits. While it is a valuable resource for the wheat research community, it is limited to wheat and covers many traits without disease-specific focus. FHBMarkerDb, in contrast, is dedicated exclusively to FHB resistance across four major crops like wheat, barley, maize, and oats offering deeper and more targeted curation of FHB-related QTLs, GWAS/MTAs, linked markers, candidate genes, and functional annotations in a single integrated platform. This focused, multi-crop integration makes it more directly useful for fine mapping, meta-QTL analysis, and marker-assisted selection for FHB resistance (Singh et al., 2021; Singh et al., 2022).

GWAS Atlas is a large multi-species database that catalogs SNP–trait associations across a wide range of plants and animals, providing tools for browsing and analyzing genome-wide association data. Although useful for broad genomic discovery, it lacks the disease-specific and crop-specific depth needed for targeted breeding work. FHBMarkerDb goes beyond simply reporting SNP trait associations by linking them with QTLs, candidate genes, and functional annotations relevant to FHB resistance across wheat, barley, maize, and oats, making it more applicable for translational research and crop improvement (Tian et al., 2020; Liu et al., 2023).

Other widely used resources, including Gramene, Ensembl Plants, and MaizeGDB, primarily focus on genome annotation, comparative genomics, or crop-specific genetic resources (Bolser et al., 2016; Gupta et al., 2016; Lawrence et al., 2004). While highly valuable for general genomic studies, they do not specifically curate FHB-related QTLs, MTAs, or integrated functional annotations across multiple cereal crops. In contrast, FHBMarkerDb uniquely integrates multi-crop FHB-associated MTAs/QTLs, candidate genes, GO terms, and pathway information into a single disease-focused platform, enabling direct application in fine mapping, functional interpretation, and marker-assisted breeding for FHB resistance.

Overall, unlike these general-purpose databases, FHBMarkerDb offers a disease-specific, multi-crop, multi-layer data integration approach that bridges the gap between statistical genomic associations and practical breeding applications for FHB resistance.

## 4 Conclusion

FHBMarkerDb compiles manually curated MTAs and QTLs associated with FHB resistance and related traits in major cereal crops, namely wheat, barley, maize, and oats. All genome-anchored markers and loci are systematically organized within a relational framework and made available to users through an interactive web interface. Comprehensive details, such as chromosomal location, associated characteristic, marker category, associated candidate genes, GO annotations, and pathway details, are given for each marker. The database offers multiple browsing and search options based on crop, chromosome, trait, and gene, allowing efficient data retrieval and cross-species comparison. By using integrated marker-trait-gene linkages, users can quickly prioritize functional candidates and find important resistance regions. FHBMarkerDb offers a valuable resource for comparative genomics, functional characterization, and genomics-assisted breeding for improved FHB resistance in cereals by combining widely dispersed genomic data into a single standardized platform.

To ensure long-term relevance, FHBMarkerDb will be updated on a regular basis to incorporate newly published GWAS results, QTL mapping studies, and marker–trait association data across all four crops. Expansion to include more diverse germplasm and multi-environment datasets is also planned to improve the reliability of identified resistance loci. Overall, FHBMarkerDb will continue to evolve as a comprehensive, curated, and interoperable platform supporting researchers, breeders, and students working on FHB resistance and cereal crop improvement.

## Data Availability

Publicly available datasets were analyzed in this study. This data can be found here: https://nbpgr.org.in/FHBDb/index.php.

## References

[B1] Akohoue F. Miedaner T. (2022). Meta-analysis and co-expression analysis revealed stable QTL and candidate genes conferring resistances to Fusarium and Gibberella ear rots while reducing mycotoxin contamination in maize. Front. Plant Sci. 13, 1050891. doi: 10.3389/fpls.2022.1050891 36388551 PMC9662303

[B2] Alisaac E. Mahlein A. K. (2023). Fusarium head blight on wheat: Biology, modern detection and diagnosis and integrated disease management. Toxins 15, 192. doi: 10.3390/toxins15030192 36977083 PMC10053988

[B3] Asiedu D. D. Miedaner T. (2025). Genetic and genomic tools in breeding for resistance to Fusarium stalk rot in maize (Zea mays L.). Plants 14, 819. doi: 10.3390/plants14050819 40094830 PMC11902793

[B4] Bolser D. M. Staines D. M. Perry E. Kersey P. J. (2016). “ Ensembl plants: integrating tools for visualizing, mining, and analyzing plant genomic data,” in Plant genomics databases: methods and protocols ( Springer New York, New York, NY), 1–31. doi: 10.1007/978-1-4939-6658-5_1 27987162

[B5] Buerstmayr M. Steiner B. Buerstmayr H. (2020). Breeding for Fusarium head blight resistance in wheat—Progress and challenges. Plant Breed. 139, 429–454. doi: 10.1111/pbr.12797 40046247

[B6] Cao A. de la Fuente M. Gesteiro N. Santiago R. Malvar R. A. Butrón A. (2022). Genomics and pathways involved in maize resistance to Fusarium ear rot and kernel contamination with fumonisins. Front. Plant Sci. 13, 866478. doi: 10.3389/fpls.2022.866478 35586219 PMC9108495

[B7] Choo T. M. Vigier B. Shen Q. Q. Martin R. A. Ho K. M. Savard M. (2004). Barley traits associated with resistance to Fusarium head blight and deoxynivalenol accumulation. Phytopathology 94, 1145–1150. doi: 10.1094/PHYTO.2004.94.10.1145 18943804

[B8] Dahl B. Wilson W. W. (2018). Risk premiums due to Fusarium Head Blight (FHB) in wheat and barley. Agric. Syst. 162, 145–153. doi: 10.1016/j.agsy.2018.01.025 38826717

[B9] Dweba C. C. Figlan S. Shimelis H. A. Motaung T. E. Sydenham S. Mwadzingeni L. . (2017). Fusarium head blight of wheat: Pathogenesis and control strategies. Crop Prot. 91, 114–122. doi: 10.1016/j.cropro.2016.10.002 38826717

[B10] Gupta P. Naithani S. Tello-Ruiz M. K. Chougule K. D’Eustachio P. Fabregat A. . (2016). Gramene database: navigating plant comparative genomics resources. Curr. Plant Biol. 7, 10–15. doi: 10.1016/j.cpb.2016.12.005 28713666 PMC5509230

[B11] Haile J. K. Sertse D. N’Diaye A. Klymiuk V. Wiebe K. Ruan Y. . (2023). Multi-locus genome-wide association studies reveal the genetic architecture of Fusarium head blight resistance in durum wheat. Front. Plant Sci. 14, 1182548. doi: 10.3389/fpls.2023.1182548 37900749 PMC10601657

[B12] Islam T. (2022). An integrated pest management program for managing fusarium head blight disease in cereals. J. Integr. Agric. 21, 3434–3444. doi: 10.1016/j.jia.2022.08.053 38826717

[B13] Kazan K. Gardiner D. M. (2018). Transcriptomics of cereal–Fusarium graminearum interactions: what we have learned so far. Mol. Plant Pathol. 19, 764–778. doi: 10.1111/mpp.12561 28411402 PMC6638174

[B14] Lanubile A. Maschietto V. Borrelli V. M. Stagnati L. Logrieco A. F. Marocco A. (2017). Molecular basis of resistance to Fusarium ear rot in maize. Front. Plant Sci. 8, 1774. doi: 10.3389/fpls.2017.01774 29075283 PMC5644281

[B15] Lawrence C. J. Dong Q. Polacco M. L. Seigfried T. E. Brendel V. (2004). MaizeGDB, the community database for maize genetics and genomics. Nucleic Acids Res. 32, D393–D397. doi: 10.1093/nar/gkh011 14681441 PMC308746

[B16] Legrand F. Chen W. Cobo-Díaz J. F. Picot A. Floch G. L. (2019). Co-occurrence analysis reveal that biotic and abiotic factors influence soil fungistasis against Fusarium graminearum. FEMS Microbiol. Ecol. 95, fiz056. doi: 10.1093/femsec/fiz056 30998232

[B17] Ma Z. Xie Q. Li G. Jia H. Zhou J. Kong Z. . (2020). Germplasms, genetics and genomics for better control of disastrous wheat Fusarium head blight. Theor. Appl. Genet. 133, 1541–1568. doi: 10.1007/s00122-019-03525-8 31900498

[B18] McMullen M. Bergstrom G. De Wolf E. Dill-Macky R. Hershman D. Shaner G. . (2012). A unified effort to fight an enemy of wheat and barley: Fusarium head blight. Plant Dis. 96, 1712–1728. doi: 10.1094/PDIS-03-12-0291-FE 30727259

[B19] Mir Z. A. Chandra T. Saharan A. Budhlakoti N. Mishra D. C. Saharan M. S. . (2023). Recent advances on genome-wide association studies (GWAS) and genomic selection (GS); prospects for Fusarium head blight research in Durum wheat. Mol. Biol. Rep. 50, 3885–3901. doi: 10.1007/s11033-023-08309-4 36826681

[B20] Nannuru V. K. R. Windju S. S. Belova T. Dieseth J. A. Alsheikh M. Dong Y. . (2022). Genetic architecture of fusarium head blight disease resistance and associated traits in Nordic spring wheat. Theor. Appl. Genet. 135, 2247–2263. doi: 10.1007/s00122-022-04109-9 35597885 PMC9271104

[B21] Neupane A. Tamburic‐llincic L. Brûlé‐Babel A. McCartney C. A. (2025). Genetic improvement of FHB and DON resistance by combining the Fhb1 gene with additional resistance QTL in winter wheat population. Plant Genome 18, e70084. doi: 10.1002/tpg2.70084 40815199 PMC12356168

[B22] Powell A. J. Vujanovic V. (2021). Evolution of Fusarium head blight management in wheat: Scientific perspectives on biological control agents and crop genotypes protocooperation. Appl. Sci. 11, 8960. doi: 10.3390/app11198960 30654563

[B23] Ren H. Zhang X. Zhang Y. Wang J. Zhang Z. Cheng M. . (2025). Identification and genome-wide association analysis of wheat FHB resistance genes. Plant Dis. 110 (2), pp.347-356. doi: 10.1094/PDIS-02-25-0298-RE 40308069

[B24] Sallam A. H. Haas M. Huang Y. Tandukar Z. Muehlbauer G. Smith K. P. . (2024). Meta‐analysis of the genetics of resistance to Fusarium head blight and deoxynivalenol accumulation in barley and considerations for breeding. Plant Breed. 143 (1), 2–25. 40046247

[B25] Santiago R. Cao A. Malvar R. A. Butrón A. (2020). Genomics of maize resistance to Fusarium ear rot and fumonisin contamination. Toxins 12, 431. doi: 10.3390/toxins12070431 32629954 PMC7404995

[B26] Song P. Li Y. Wang X. Wang X. Zhang A. Wang Z. . (2025). Exploration of genomic regions associated with fusarium head blight resistance in wheat and development and validation of kompetitive allele-specific polymerase chain reaction markers. Int. J. Mol. Sci. 26, 3339. doi: 10.3390/ijms26073339 40244225 PMC11989977

[B27] Su Z. Jin S. Zhang D. Bai G. (2018). Development and validation of diagnostic markers for Fhb1 region, a major QTL for Fusarium head blight resistance in wheat. Theor. Appl. Genet. 131, 2371–2380. doi: 10.1007/s00122-018-3159-6 30136107

[B28] Venske E. Dos Santos R. S. Farias D. D. R. Rother V. Da Maia L. C. Pegoraro C. . (2019). Meta-analysis of the QTLome of Fusarium head blight resistance in bread wheat: refining the current puzzle. Front. Plant Sci. 10, 727. doi: 10.3389/fpls.2019.00727 31263469 PMC6585393

[B29] Viviani A. Haile J. K. Fernando W. D. Ceoloni C. Kuzmanović L. Lhamo D. . (2025). Priority actions for Fusarium head blight resistance in durum wheat: Insights from the wheat initiative. Plant Genome 18, e20539. doi: 10.1002/tpg2.20539 39757924 PMC11701714

[B30] Wilson W. Dahl B. Nganje W. (2018). Economic costs of Fusarium Head Blight, scab and deoxynivalenol. World Mycotoxin J. 11, 291–302. doi: 10.3920/wmj2017.2204

[B31] Zhang F. Zhang H. Liu J. Ren X. Ding Y. Sun F. . (2024). Fhb9, a major QTL for Fusarium head blight resistance improvement in wheat. J. Integr. Agric. doi: 10.1016/j.jia.2024.03.045 38826717

[B32] Zheng T. Hua C. Li L. Sun Z. Yuan M. Bai G. . (2021). Integration of meta-QTL discovery with omics: Towards a molecular breeding platform for improving wheat resistance to Fusarium head blight. Crop J. 9 (4), 739–749. 38826717

[B33] Zhu Z. Zhu X. Zhang N. Wang W. Liu J. Zhang F. . (2025). Identification and validation of a major QTL, QFhb-6AL, for Fusarium head blight resistance on chromosome 6AL in wheat. Theor. Appl. Genet. 138, 74. doi: 10.1007/s00122-025-04864-5 40089629

