# Supplementary material for: FHBMarkerDb: unifying genomic markers and functional annotations for durable FHB resistance in major cereals
Source: Front Plant Sci. 2026 Jul 16;17:1846092. doi: 10.3389/fpls.2026.1846092 (PMC13422196; doi:10.3389/fpls.2026.1846092)
Supplement: Supplementary file 1 [file DataSheet1.pdf]

Fig S1. Step-wise workflow of the first application case in FHBMarkerDb.

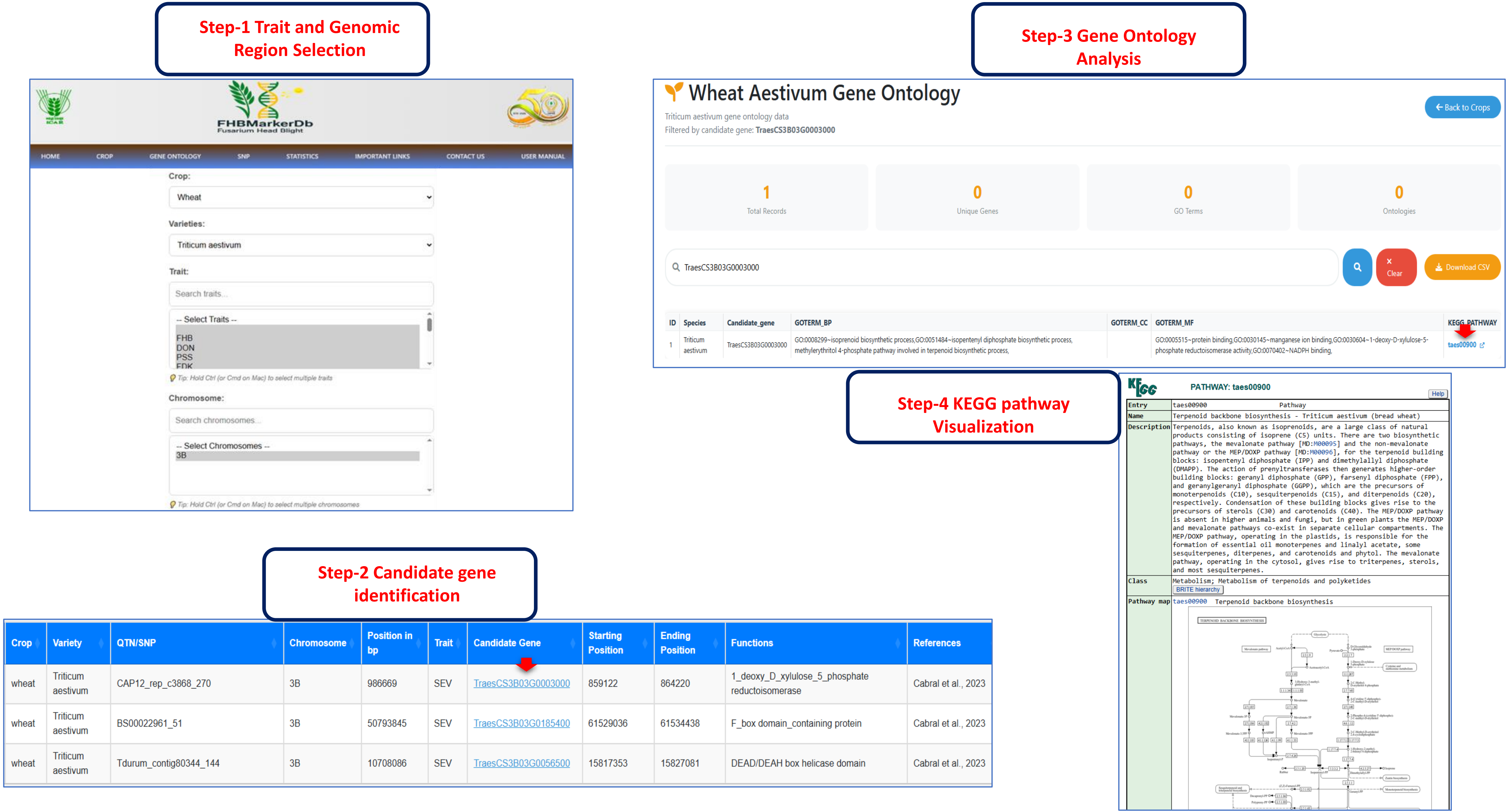

Crop	Variety	QTN/SNP	Chromosome	Position in bp	Trait	Candidate Gene	Starting Position	Ending Position	Functions	References
wheat	Triticum aestivum	CAP12_rep_c3868_270	3B	986669	SEV	TraesCS3B03G0003000	859122	864220	1_deoxy_D_xylulose_5_phosphate reductoisomerase	Cabral et al., 2023
wheat	Triticum aestivum	BS00022961_51	3B	50793845	SEV	TraesCS3B03G0185400	61529036	61534438	F_box domain_containing protein	Cabral et al., 2023
wheat	Triticum aestivum	Tdurum_contig80344_144	3B	10708086	SEV	TraesCS3B03G0056500	15817353	15827081	DEAD/DEAH box helicase domain	Cabral et al., 2023
